# Supplementary material for: Distinct manifestations of excitatory-inhibitory imbalance associated with amyloid-β and tau in patients with Alzheimer’s disease
Source: Nat Commun. 2025 Aug 26;16:7957. doi: 10.1038/s41467-025-62798-4 (PMC12381375; doi:10.1038/s41467-025-62798-4)
Supplement: Supplementary file 1 — Supplementary Information [file 41467_2025_62798_MOESM1_ESM.pdf]

SUPPLEMENT

1. SUPPLEMENTARY FIGURES

1.1. **Figure S1: Associations between neural excitability and neural fragility with Aβ and tau accumulations with CDR included as a categorical covariate.**

1.2. **Figure S2: Associations between neural excitability and neural fragility with tau accumulation after covarying Aβ accumulation.**

1.3. **Figure S3: Neural excitability and neural fragility associations with hypometabolism with CDR included as a categorical covariate.**

1.4. **Figure S4: Neural fragility and neural excitability associations with hypometabolism after controlling for cortical atrophy.**

1.5. **Figure S5: Neural excitability and neural fragility in AD-EPI+ and AD-EPI− patients compared to healthy controls.**

2. SUPPLEMENTARY TABLES

2.1. **Table S1: Neuropsychological test performance for AD patients**

2.2. **Table S2: Demographics of each imaging sub-cohort of AD patients**

2.3. **Table S3: Demographic and clinical characterization of AD-EPI+ and AD-EPI− patients**

1. SUPPLEMENTARY FIGURES

1.1. Figure S1: Associations between neural excitability and neural fragility with Aβ and tau accumulations with CDR included as a categorical covariate.

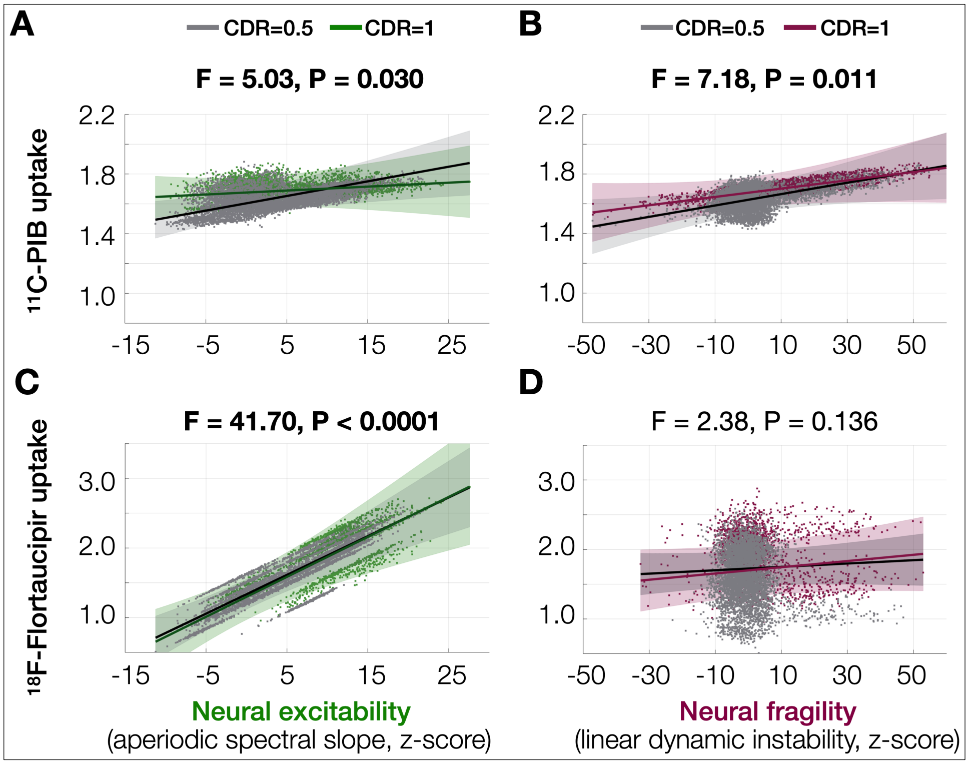

Figure S1: Linear mixed model (LMM) analyses, including the additional categorical variable of CDR revealed similar associations in each CDR subgroup between Aβ accumulation and measures of E/I imbalance. Higher Aβ is positively correlated with both neural excitability (aperiodic spectral slope; A), and with neural fragility (linear dynamic instability; B). There were no significant interactions with CDR group and neural excitability (F=1.67, P=0.2027) or with neural fragility (F=0.17, P=0.6814). Likewise, the associations between regional tau accumulation and measures of E/I imbalance showed similar patterns in CDR=0.5 and CDR=1. In both CDR subgroups, higher tau accumulation showed a strong positive correlation with neural excitability (C) but was not correlated with neural fragility (D). There were no significant interactions with CDR group and neural excitability (F=0.01, P=0.9193) or with neural fragility (F=0.21, P=0.6502). The dark lines in each subplot indicate the model predictions within each CDR subgroup and the shaded areas indicate 95% confidence limits. Abbreviations: CDR, Clinical Dementia Rating; E/I, excitatory-to-inhibitory; LMM, linear mixed model; PIB, Pittsburgh compound B.

1.2. Figure S2: Associations between neural excitability and neural fragility with tau accumulation after covarying Aβ accumulation.

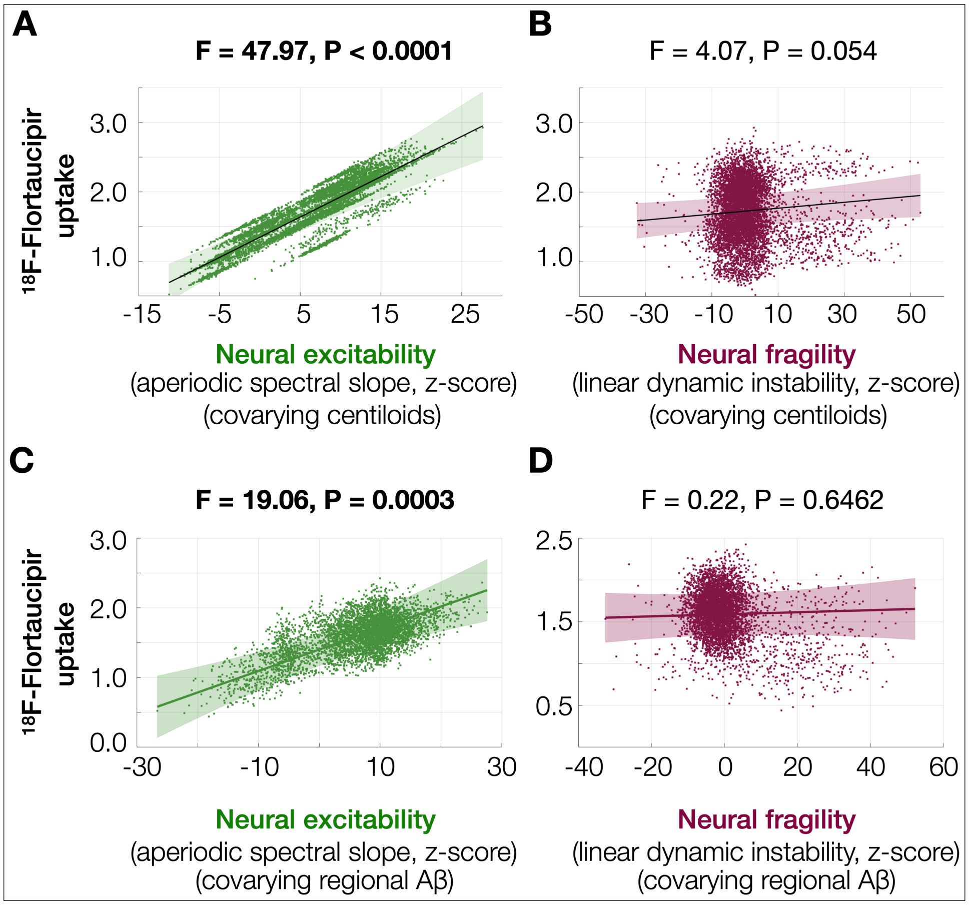

Figure S2: Linear mixed model (LMM) analyses showed the same associations between tau accumulation and measures of E/I imbalance after covarying Aβ. In an LMM which included global Aβ in centiloid scale in the same sub-cohort of 35 AD patients imaged with MEG and flortaucipir, higher tau accumulation positively correlated with higher neural excitability (aperiodic spectral slope; A), but not with neural fragility (linear dynamic instability; B). In an LMM which included the sub-cohort of AD patients (n=23) who were uniformly imaged with ^{11}C-PIB as the Aβ-PET tracer in addition to flortaucipir as the tau-PET tracer, regional Aβ SUVR values were included as a covariate into the LMM, which showed that positive associations between tau accumulation and higher neural excitability were still statistically significant (C), while no significant associations were found between tau and neural fragility (D). The dark line in each subplot indicates the model predictions and the shaded area indicates 95% confidence limits. Abbreviations: E/I, excitatory-to-inhibitory; LMM, linear mixed model; PIB, Pittsburgh compound B.

1.3. Figure S3: Neural excitability and neural fragility associations with hypometabolism with CDR included as a categorical covariate.

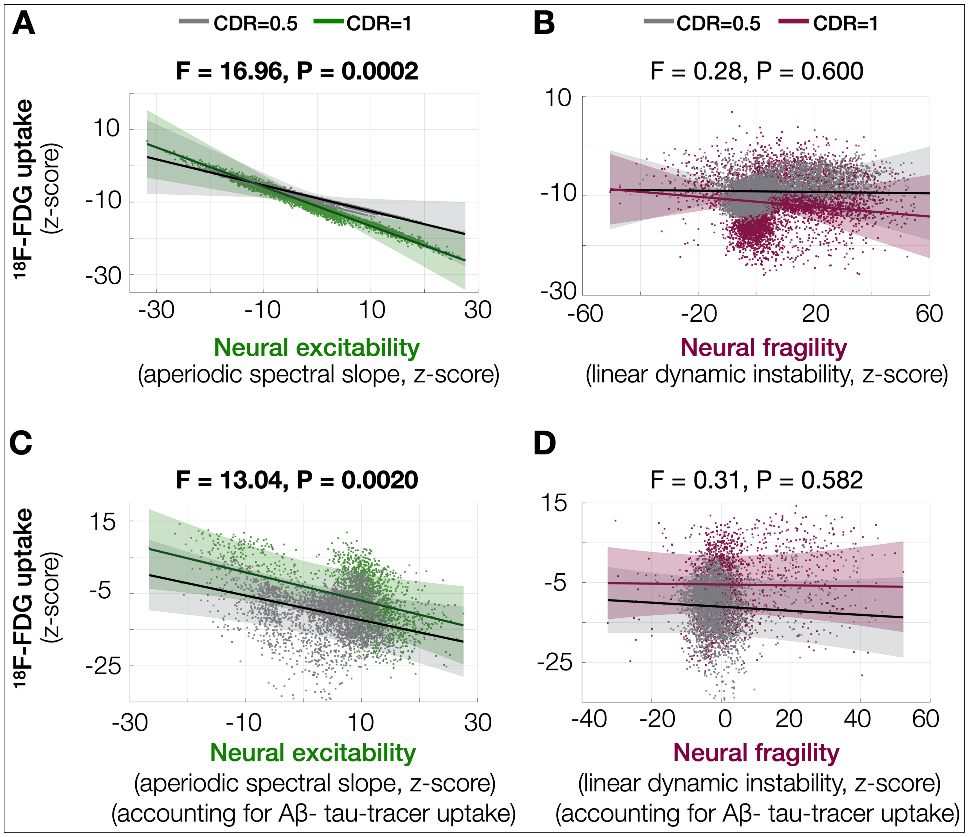

Figure S3: Linear mixed model (LMM) analyses, including the additional categorical variable of CDR, revealed similar associations in each CDR subgroup between hypometabolism (FDG-PET) and measures of E/I imbalance. In both CDR groups, greater hypometabolism (lower FDG-PET) correlated with higher neural excitability (aperiodic spectral slope; A), but not with neural fragility (linear dynamic instability; B). There were no significant interactions with CDR group and neural excitability (F=0.71, P=0.4050) or with neural fragility (F=0.16, P=0.6878). The associations between hypometabolism (FDG-PET) and measures of E/I imbalance, after being corrected for regional Aβ and tau uptakes also showed similar patterns in CDR=0.5 and CDR=1. In both CDR subgroups, greater hypometabolism correlated with higher neural excitability (C) but was not correlated with neural fragility (D). There were no significant interactions with CDR group and neural excitability (F=0.06, P=0.8071) or with neural fragility (F=0.13, P=0.7198). The dark lines in each subplot indicate the model predictions within each CDR subgroup and the shaded areas indicate 95% confidence limits. Abbreviations: CDR, Clinical Dementia Rating; E/I, excitatory-to-inhibitory; LMM, linear mixed model; PIB, Pittsburgh compound B.

1.4. Figure S4: Neural fragility and neural excitability associations with hypometabolism after controlling for cortical atrophy.

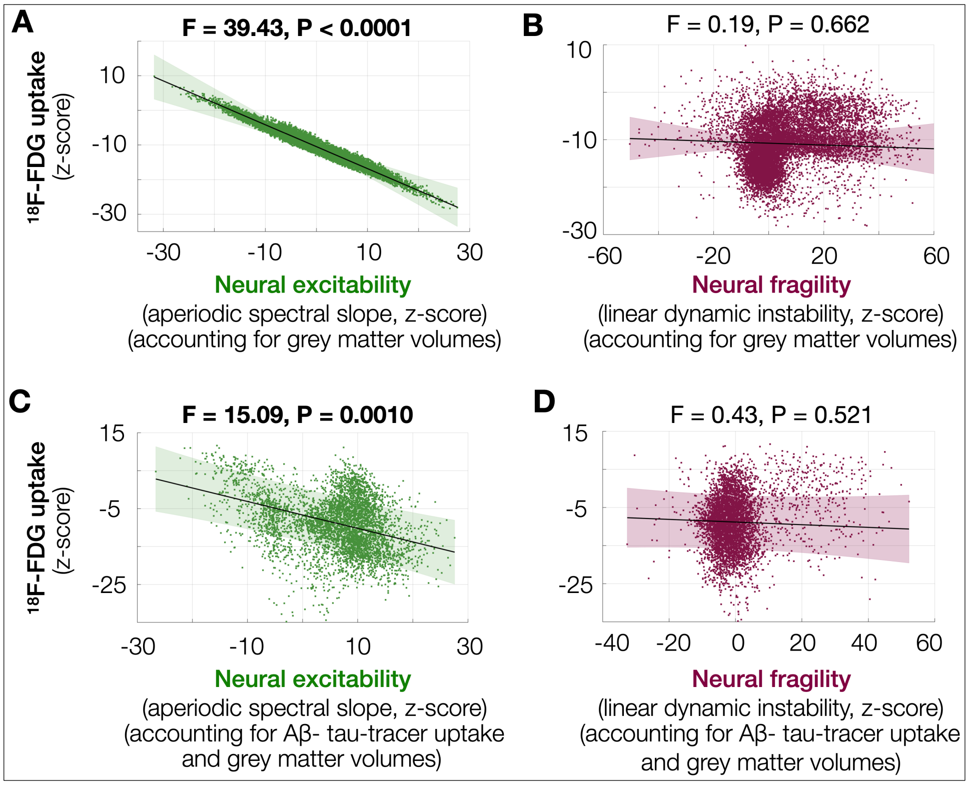

Figure S4: In AD patients who were imaged with MEG and FDG-PET (n=53), a linear mixed model (LMM) analysis, including cortical grey matter volume as a covariate, showed that higher neural excitability is strongly correlated with greater hypometabolism (B), while neural fragility did not show significant associations (A). In AD patients who were imaged with MEG and triple-PET (Aβ, tau, and FDG) imaging (n=23), an LMM, including cortical grey matter volume as a covariate, showed that higher neural excitability was significantly correlated with hypometabolism even after covarying the effects of Aβ and tau (D), while neural fragility remained uncorrelated (C). The dark line indicates the model prediction, and the shaded area indicates the 95% confidence limits. The scatter plots show each individual subject's data from 210 cortical regions. The models include random intercepts and random slopes with brain regions incorporated as repeated measures.

1.5. Figure S5: Neural excitability and neural fragility in AD-EPI+ and AD-EPI− patients compared to healthy controls.

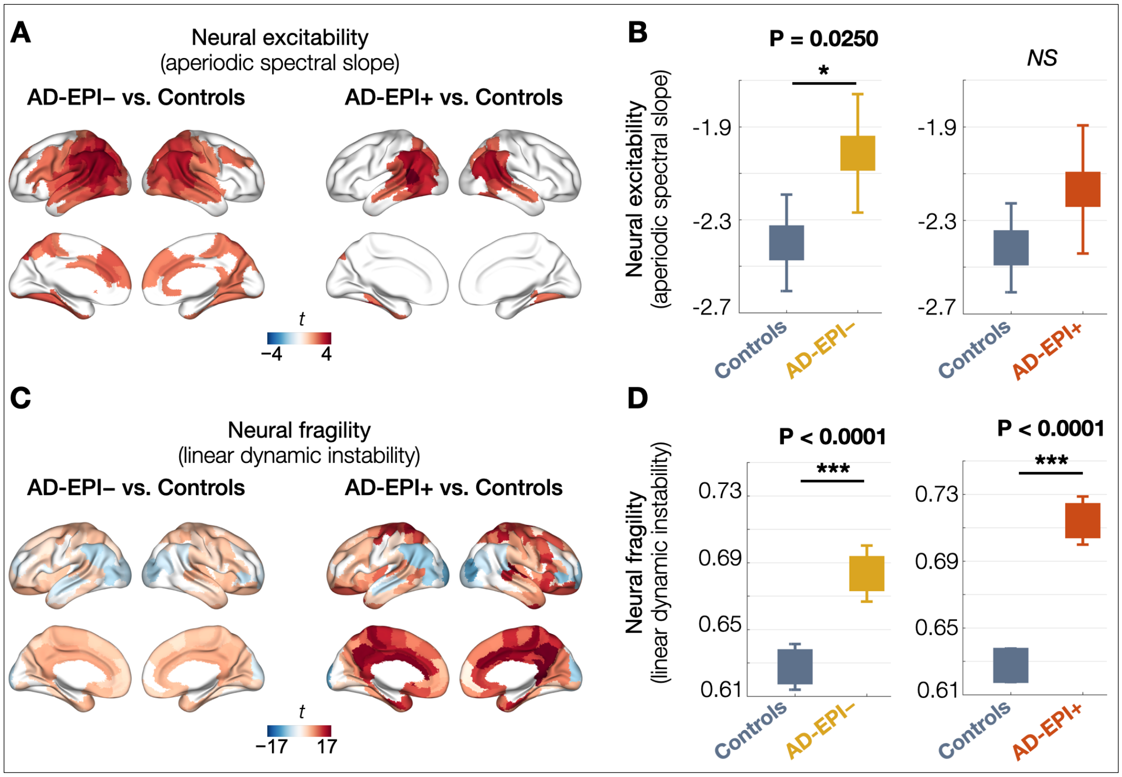

Figure S5: Neural excitability (aperiodic spectral slope) in AD-EPI− compared to controls was increased in posterior temporoparietal, inferior temporal, and medial dorsal regions of the frontal cortices, while increased neural excitability in AD-EPI+ vs. controls showed a less extensive distribution restricted to temporoparietal cortex (A). An LMM with repeated measures showed significantly higher neural excitability in AD-EPI− vs. controls (t=2.30, P=0.0250), but no statistically significant difference between AD-EPI+ and controls (t=1.47, P=0.1475). Neural fragility (linear dynamic instability) in AD-EPI− and also AD-EPI+ compared to controls showed spatial patterns of increased fragility over lateral frontal and central cortices as well as medial cortices of the brain, where AD-EPI+ showed stronger effects (C). An LMM with repeated measures showed significantly higher neural fragility in AD-EPI− vs. controls (t=5.05, P<0.0001), as well as between AD-EPI+ vs. controls (t=9.63, P<0.0001). Brain renderings in C-D show statistical significance from group comparison after covarying age and thresholded at FDR 10%. The subplots in B and D denote each group's least square means and standard error. Abbreviations: AD, Alzheimer's disease; FDR, false discovery rate; LMM, linear mixed model.

2. SUPPLEMENTARY TABLES

2.1. Table S1: Neuropsychological test performance for AD patients

Variable	Mean ± SD	Range (Min-Max)	N
Episodic memory function			
Visual free recall (Benson 10 minutes)	5.38 ± 4.20	0 - 15	78
Short delay verbal memory (CVLT 30 seconds)	3.87 ± 2.47	0 - 9	77
Verbal free recall (CVLT 10 minutes)	2.42 ± 2.75	0 - 9	77
Executive function & working memory			
Design Fluency	6.31 ± 3.67	0 - 18	71
Information processing speed (Stroop color naming)	48.45 ± 23.65	2 - 112	64
Cognitive control (Stroop Inhibition)	22.26 ± 15.73	0 - 66	58
Verbal working memory (Digit span forward)	5.05 ± 1.32	0 - 8	81
Attention (Digit span backward)	3.32 ± 1.34	0 - 7	81
Set shifting (Modified trails – speed)	0.22 ± 0.20	0.01 - 0.70	63
Verbal learning (CVLT total score)	16.83 ± 6.6	5 - 32	77
Language function			
Reading irregular words	5.65 ± 0.66	4 - 6	60
Syntax comprehension	3.61 ± 1.34	1 - 5	75
Verbal Agility	4.37 ± 1.73	0 - 6	76
Boston Naming Test	11.7 ± 3.16	1 - 15	76
Lexical Fluency (D words/1 minute)	9.74 ± 5.25	0 - 26	80
Category Fluency (Animals/1 minute)	11.8 ± 6.44	2 - 33	80
Repetition	3.37 ± 1.47	0 - 5	79
Visuospatial function			
Face discrimination (CATS – face matching)	10.92 ± 1.46	6 - 12	66
Visuoconstruction (Benson copy)	11.74 ± 4.86	0 - 16	78
Location discrimination (VOSP number location)	6.79 ± 2.82	1 - 10	73
Calculations	3.08 ± 1.43	0 - 5	78
Emotion naming (CATS – affect matching)	11.9 ± 1.81	6 - 15	61

Abbreviations: CATS, Comprehensive Affect Testing System; CDR, Clinical Dementia Rating; CDR-SOB, CDR Sum of Boxes; CVLT, California Verbal Learning Test containing 9 items; MMSE, Mini-Mental State Examination; VOSP, Visual Object and Space Perception.

2.2. Table S2: Demographics of each imaging sub-cohort of AD patients

	AD patients scanned with ^{11}C-PIB (Aβ-PET) (n=52)	AD patients scanned with flortaucipir (tau-PET) (n=35)	AD patients scanned with ^{18}F-FDG (FDG-PET) (n=51)	AD patients scanned with triple PET imaging (n=23)
Age – yr	62.79 ± 1.19	63.50 ± 1.56	61.51 ± 1.17	63.53 ± 2.02
Female sex – no. (%)[a]	31 (59.61)	19 (54.29)	30 (58.82)	12 (52.17)
Right handedness – no. (%)	46 (88.46)	30 (85.71)	43 (84.31)	20 (86.95)
Education – yr	16.73 ± 0.38	17.03 ± 0.49	16.96 ± 0.37	17.78 ± 0.59
CDR[b]	0.72 ± 0.034	0.66 ± 0.04	0.77 ± 0.04	0.70 ± 0.05
Time from MEG scan (months)	4.27 ± 1.39[c]	2.50 ± 1.03[d]	5.80 ± 1.78[e]	-

[a] Sex was self-reported.

[b] Scores on the CDR range from 0 to 3 with higher scores denoting greater impairment. Values shown for age, education, CDR and time from MEG scan indicate Mean ± SE.

[c] 46 out of 52 AD patients imaged with ^{11}C-PIB were imaged within 12 months from the MEG scan and the rest of the 6 patients were imaged within 32 months.

[d] 33 out of 35 AD patients imaged with flortaucipir were imaged within 12 months from the MEG scan and the remaining 2 patients were scanned within 24 months.

[e] 43 out of 51 AD patients scanned with FDG-PET were imaged within 12 months from the MEG scan and 7 of the remaining patients were scanned within 32 months; one patient was scanned within 55 months from the MEG scan.

Abbreviations: AD, Alzheimer's disease; CDR, Clinical Dementia Rating.

2.3. Table S3: Demographic and clinical characterization of AD-EPI+ and AD-EPI− patients

Characteristic	AD-EPI− (N=27)	AD-EPI+ (N=20)
Age – yr	60.7 ± 8.3	59.9 ± 6.7
Female sex – no. (%)[a]	17 (56.7)	12 (60.0)
White – no. (%)[b]	27 (100)	19 (100.0)
Education – yr	15.7 ± 2.6	17.0 ± 2.7
Right handedness – no. (%)	25 (83.3)	18 (90.0)
Apo E ε4 carrier – no. (%)	12 (44.4)	9 (47.4)
MMSE	21.3 ± 5.8	21.5 ± 4.7
CDR	1.0 (0.5 – 1.0)	1.0 (0.5 – 1.0)
CDR-SOB	4.5 (3.5 – 5.0)	4.7 (3.3 – 6.5)
Age at disease onset	54.0 (50.0 -58.0)	53.5 (50.0 – 57.5)
Disease duration	5.4 (4.7 – 7.2)	4.8 (3.8 – 6.5)
Early onset AD – no. (%)	28 (93.3)	19 (95.0)
Atypical AD – no. (%)	11 (36.7)	7 (35.0)
Generalized slowing on LTM-EEG – no. (%)	7 (23.3)	6 (30.0)
Asymmetric / focal slowing on LTM-EEG – no. (%)	4 (13.3)	3 (15.0)
Generalized slowing on MEG-EEG – no. (%)	13 (43.3)	6 (30.0)
Asymmetric / focal slowing on MEG-EEG – no. (%)	5 (16.7)	8 (40.0)
On AChE-I – no. (%)	15 (50.0)	12 (60.0)
On memantine – no. (%)	1 (3.3)	1 (5.0)
On AChE-I & memantine – no. (%)	7 (23.3)	3 (15.0)
On antidepressants – no. (%)	15 (50.0)	11 (55.0)

[a] Sex was self-reported.

[b] Race or ethnic group was self-reported; one AD-EPI+ patient opted out of reporting the race.

Abbreviations: AChE-I, Acetylcholinesterase inhibitor; AD, Alzheimer's disease; AD-EPI−, AD patients without epileptiform activity; AD-EPI+, AD patients with epileptiform activity; ApoE, Apolipoprotein E; CDR, Clinical Dementia Rating; CDR-SOB, CDR-Sum of Boxes; EEG, electroencephalography; MEG, magnetoencephalography; MMSE, Mini Mental State Examination.
