# Supplementary material for: Distinct manifestations of excitatory-inhibitory imbalance associated with amyloid-β and tau in patients with Alzheimer’s disease
Source: Nat Commun. 2025 Aug 26;16:7957. doi: 10.1038/s41467-025-62798-4 (PMC12381375; doi:10.1038/s41467-025-62798-4)
Supplement: Supplementary file 2 — Reporting Summary [file 41467_2025_62798_MOESM2_ESM.pdf]

nature portfolio

Corresponding author(s): Kamalini G Ranasinghe

Last updated by author(s): Jun 4, 2025

Reporting Summary

Nature Portfolio wishes to improve the reproducibility of the work that we publish. This form provides structure for consistency and transparency in reporting. For further information on Nature Portfolio policies, see our Editorial Policies and the Editorial Policy Checklist.

Statistics

For all statistical analyses, confirm that the following items are present in the figure legend, table legend, main text, or Methods section.

n/a	Confirmed	
☐	☒	The exact sample size (n) for each experimental group/condition, given as a discrete number and unit of measurement
☐	☒	A statement on whether measurements were taken from distinct samples or whether the same sample was measured repeatedly
☐	☒	The statistical test(s) used AND whether they are one- or two-sided<br>*Only common tests should be described solely by name; describe more complex techniques in the Methods section.*
☐	☒	A description of all covariates tested
☐	☒	A description of any assumptions or corrections, such as tests of normality and adjustment for multiple comparisons
☐	☒	A full description of the statistical parameters including central tendency (e.g. means) or other basic estimates (e.g. regression coefficient) AND variation (e.g. standard deviation) or associated estimates of uncertainty (e.g. confidence intervals)
☐	☒	For null hypothesis testing, the test statistic (e.g. F, t, r) with confidence intervals, effect sizes, degrees of freedom and P value noted<br>*Give P values as exact values whenever suitable.*
☒	☐	For Bayesian analysis, information on the choice of priors and Markov chain Monte Carlo settings
☒	☐	For hierarchical and complex designs, identification of the appropriate level for tests and full reporting of outcomes
☐	☒	Estimates of effect sizes (e.g. Cohen's d, Pearson's r), indicating how they were calculated

Our web collection on statistics for biologists contains articles on many of the points above.

Software and code

Policy information about availability of computer code

Data collection	Each subject underwent a minimum of 10 minutes MEG recording on a whole-head biomagnetometer system consisting of 275 axial gradiometers (OMEGA 275, CTF Inc. Port Coquitlam, British Columbia, Canada). The Spike Density Calculation Engine in Persyst-11 EEG software was used to help detect epileptiform activity on the LTM-EEG.All PIB-PET scans were acquired at LBNL on a Siemens Biograph 6 Truepoint PET/CT scanner (Siemens Medical Systems) in 3D acquisition mode.All FDG-PET, scans were acquired at LBNL, including 39 scans on a Siemens Biograph 6 Truepoint PET/CT scanner (Siemens Medical Systems; in 3D acquisition mode) and 12 scans on ECAT EXACT HR (3D acquisition mode). All flortaucipir PET scans were acquired on a Siemens Biograph PET/CT scanner (Siemens Medical Systems) in 3D acquisition mode.
Data analysis	Data analyses utilized Matlab -2020 and Matlab-2023; SPM12 toolbox, and SAS-version 9. We did not use open source-codes. All analyses were performed using custom scripts based on established Matlab and SAS functions included in the software packages.

For manuscripts utilizing custom algorithms or software that are central to the research but not yet described in published literature, software must be made available to editors and reviewers. We strongly encourage code deposition in a community repository (e.g. GitHub). See the Nature Portfolio guidelines for submitting code & software for further information.

Data

Policy information about availability of data

All manuscripts must include a data availability statement. This statement should provide the following information, where applicable:

- Accession codes, unique identifiers, or web links for publicly available datasets
- A description of any restrictions on data availability
- For clinical datasets or third party data, please ensure that the statement adheres to our policy

Data and materials availability: All data associated with this study are presented in the main content and supplemental information sections. Source data for figures are provided with this paper. Deidentified imaging (MEG and MRI) data are saved in the OSF and are publicly available. (https://osf.io/pd4h9/files/osfstorage). Deidentified PET and clinical data will be shared on request from qualified investigators for the purposes of replicating procedures and results, and for other non-commercial research purposes within the limits of participants' consent. Correspondence and material requests should be addressed to Kamalini.ranasinghe@ucsf.edu. Code availability: All custom scripts used for data analysis in this study have been deposited on a GitHub repository (https://github.com/kamalinigr/megabtaufdg).

Research involving human participants, their data, or biological material

Policy information about studies with human participants or human data. See also policy information about sex, gender (identity/presentation), and sexual orientation and race, ethnicity and racism.

Reporting on sex and gender	We report the group level sex (frequency/percentage) of all participants under different cohorts in the main table and in supplementary tables. We do not separate our main findings based on sex because we do not predict that the effects explored in this study is significantly different between males and females based on previous evidence from our group as well as from others.
Reporting on race, ethnicity, or other socially relevant groupings	Race is self reported in this study. We present the group level (frequency/percentage) of race for our cohorts in the main table and in the supplementary tables. These values maybe of use for future meta-analyses and also to correctly describe the clinical populations investigated in the current study.
Population characteristics	This study included 82 patients with AD (mean age: 62.3 +/- 8.9) and 40 elderly controls (mean age: 64.0 +/- 5.2). All patients were confirmed with AD bio-markers.
Recruitment	Patients were recruited from the UCSF Memory and Aging Center. Controls were recruited from the community. Being a specialized center for dementia including frontotemporal our clinical cohorts may represent a relatively higher fraction of patients who are young onset compared to contemporary neurology clinical diagnosing dementia. The community dwelling elderly control population represents the ethnic and social demographics of the San Francisco Bay-area.
Ethics oversight	UCSF-IRB

Note that full information on the approval of the study protocol must also be provided in the manuscript.

Field-specific reporting

Please select the one below that is the best fit for your research. If you are not sure, read the appropriate sections before making your selection.

☒ Life sciences ☐ Behavioural & social sciences ☐ Ecological, evolutionary & environmental sciences

For a reference copy of the document with all sections, see nature.com/documents/nr-reporting-summary-flat.pdf

Life sciences study design

All studies must disclose on these points even when the disclosure is negative.

Sample size	We used all available data that was uniformly collected as per our analyses, based on the study criteria, from the patients and controls that were evaluated under research visits at the UCSF Memory and Aging Center. There were no apriori sample size calculation for this study. The study design was clinical observational (not a clinical trial), and did not necessitate an apriori sample size estimation. We included all available participant data in an unbiased approach in our analyses.
Data exclusions	No data were excluded
Replication	The EEG/MEG clinical readouts were independently assessed by three blinded reviewers with expertise in clinical neurophysiology. All analysis procedures were implemented using standardized, version-controlled scripts in MATLAB and SAS, with fixed random seeds applied where applicable to ensure statistical reproducibility. Detailed documentation of preprocessing steps, parameter settings, and software versions is provided to ensure transparency. All processing scripts, custom analysis code, and processed data will be made publicly available upon publication to facilitate independent verification and reuse. Cross-validation procedures and internal consistency checks were also employed to confirm the robustness of key results.
Randomization	Subjects who were positive for AD bio-markers and CDR =0.5 or 1 were included as AD patients. Subjects who were CDR=0 with no cognitive

Randomization	impairment were include as controls. The study design is clinical observational for this investigation and therefore a randomization step is not needed.
Blinding	Blinding at the point of study entry was not applicable, as participant recruitment required knowledge of clinical diagnosis to ensure appropriate group assignment (AD patients vs. controls). However, to minimize potential bias in downstream analyses, all data preprocessing and quantitative analyses were conducted blinded to group identity. Investigators performing data cleaning, feature extraction, and statistical modeling operated on anonymized datasets with group labels masked until the final stage of group-level comparisons.

Reporting for specific materials, systems and methods

We require information from authors about some types of materials, experimental systems and methods used in many studies. Here, indicate whether each material, system or method listed is relevant to your study. If you are not sure if a list item applies to your research, read the appropriate section before selecting a response.

Materials & experimental systems

n/a	Involved in the study
☒	☐ Antibodies
☒	☐ Eukaryotic cell lines
☒	☐ Palaeontology and archaeology
☒	☐ Animals and other organisms
☒	☐ Clinical data
☒	☐ Dual use research of concern
☒	☐ Plants

Methods

n/a	Involved in the study
☒	☐ ChIP-seq
☒	☐ Flow cytometry
☒	☐ MRI-based neuroimaging

Plants

Seed stocks	*Report on the source of all seed stocks or other plant material used. If applicable, state the seed stock centre and catalogue number. If plant specimens were collected from the field, describe the collection location, date and sampling procedures.*
Novel plant genotypes	*Describe the methods by which all novel plant genotypes were produced. This includes those generated by transgenic approaches, gene editing, chemical/radiation-based mutagenesis and hybridization. For transgenic lines, describe the transformation method, the number of independent lines analyzed and the generation upon which experiments were performed. For gene-edited lines, describe the editor used, the endogenous sequence targeted for editing, the targeting guide RNA sequence (if applicable) and how the editor was applied.*
Authentication	*Describe any authentication procedures for each seed stock used or novel genotype generated. Describe any experiments used to assess the effect of a mutation and, where applicable, how potential secondary effects (e.g. second site T-DNA insertions, mosiacism, off-target gene editing) were examined.*
